# Case Report: Hepatopulmonary syndrome as the first clinical manifestation of cirrhosis in a patient with underlying chronic lung disease

**DOI:** 10.12688/f1000research.15434.2

**Published:** 2019-05-16

**Authors:** Charles Murphy, Danit Arad

**Affiliations:** 1 Internal Medicine, Montefiore Medical Center, Bronx, Bronx, NY, 10029, USA

**Keywords:** Hepatopulmonary Syndrome, COPD, MAI Infection, Bronchiectasis

## Abstract

An 86 year old woman with multiple chronic lung diseases (including chronic obstructive pulmonary disease, bronchiectasis, and untreated mycobacterium avium-intracellulare) presented with two weeks of increased shortness of breath, notably worse when seated as compared to when lying down. After treatments focused on her known conditions did not resolve her dyspnea, the differential diagnosis was broadened and she was found to have evidence of cirrhosis on imaging. As a result of this new diagnosis, transthoracic echocardiography and arterial blood gas analysis were performed and together yielded the diagnosis of hepatopulmonary syndrome. We describe a rare presentation of hepatopulmonary syndrome manifesting as a patient’s first clinical evidence of suspected cirrhosis, a diagnosis made difficult by this patient’s numerous other lung diseases which muddied the picture.

## Introduction

The triad of chronic liver disease, hypoxemia, and microvascular dilatations/malformations in the lungs make up hepatopulmonary syndrome (HPS), a well-known sequela of hepatic cirrhosis ^1^. These aforementioned vascular abnormalities result in ventilation-perfusion mismatch, which yields the clinical findings of dyspnea (universally) as well as digital clubbing and/or cyanosis (both very common) ^2^. We report a case of a patient presenting with HPS as their first clinical manifestation of suspected cirrhosis, and describe how it was distinguishable from other potential causes of her symptoms such as her comorbid chronic obstructive pulmonary disease (COPD), bronchiectasis, and mycobacterium avium-intracellulare (MAI) infection.

## Case report

**Figure 1.**
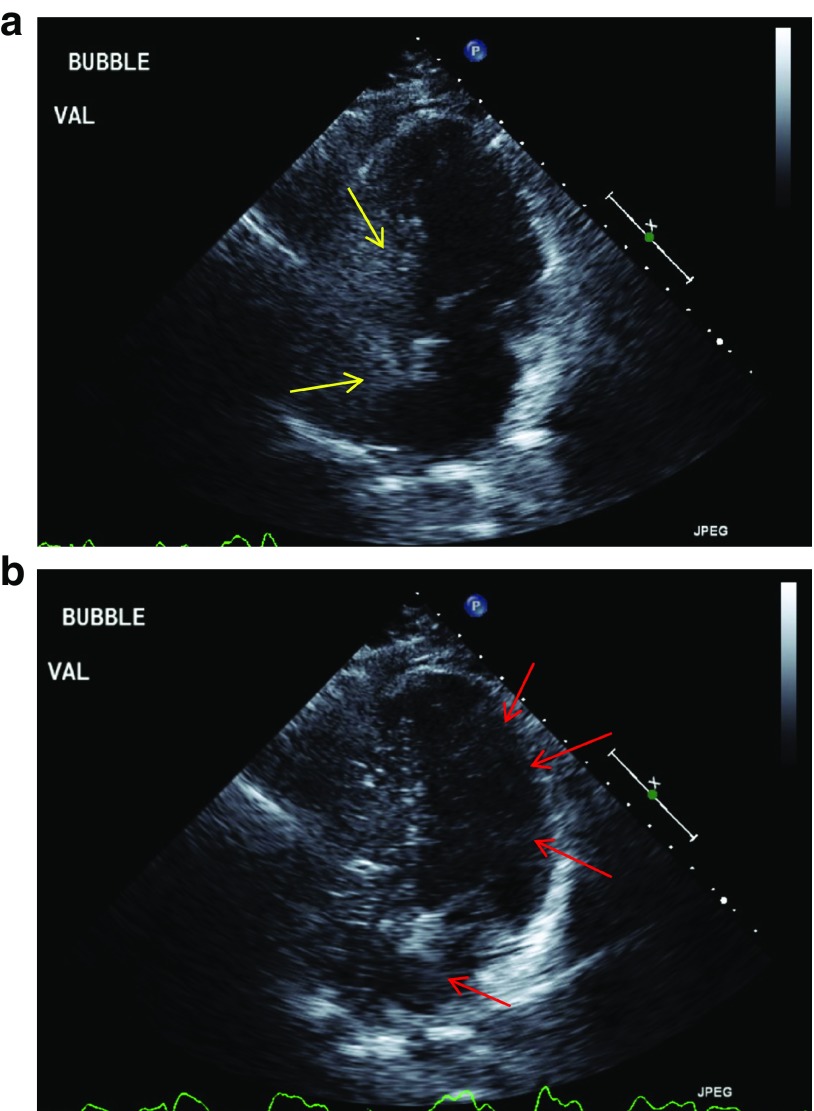
Still images from patient’s transthoracic echocardiogram showing ( **a**) no early shunting with saline bubble (identified by yellow arrows) injection, followed by ( **b**) late passage of bubbles (identified by red arrows) into the Left Atrium and Ventricle representing Intrapulmonary Shunting.

An 86 year old retired latina woman with a past medical history of COPD, bronchiectasis, MAI infection (not previously treated), tobacco dependence (40 pack-years, quit 25 years prior to presentation), diabetes mellitus, hyperlipidemia, and hypertension presented with two weeks of worsened dyspnea and non-productive cough. She reported a baseline of daily shortness of breath with an exercise tolerance of 3 blocks, but over the two weeks prior to her presentation it decreased to the point where she would feel dyspneic when walking around her apartment. Interestingly she stated that she also generally felt more short of breath while seated than when lying down, and also cited a worsening cough over this time course productive of green sputum. Her exam on presentation was significant for an oral temperature of 101.4 degrees Fahrenheit, oxygen saturation of 84% on room air, tachypnea and coarse crackles appreciated diffusely on lung examination. Her blood-work was notable for a white blood cell count of 19.8 k/µL, with multiple diffuse small nodular opacities seen on chest x-ray consistent with her known MAI infection. She was started on levofloxacin for treatment of a presumed bronchiectasis flare along with oxygen therapy via nasal cannula in addition to other supportive treatments. Although her fever, leukocytosis, and cough improved with antibiotics (further supporting a diagnosis of bronchiectasis flare), her dyspnea and hypoxemia persisted. Consequently, a chest computerized tomography (CT) scan was ordered which showed the same nodularities seen on chest x-ray, but also elucidated a nodular liver consistent with cirrhosis. While her platelet count, transaminases, bilirubin, and prothrombin time were all normal and she had no ascites or other edema on exam, she did however have spider angiomas. Further chart review done at that time revealed that she had known cirrhotic characteristics on liver imaging as they were incidentally seen almost five years prior, although she had never had any decompensations or serologic evidence of liver dysfunction since. Work-up back then elucidated no potential cause except for non-alcoholic fatty liver disease, given her histories of hyperlipidemia and diabetes. In light of this knowledge gained from deep chart review, the specter of hepatopulmonary syndrome was raised as a possible explanation for her persistent hypoxemia and dyspnea. In order to investigate this possibility, both seated and supine arterial blood gases were obtained which elucidated orthodeoxia (see Table 1). A transthoracic echocardiogram with bubble study was then performed which suggested an intrapulmonary shunt (see Figure 1), thereby confirming the diagnosis of HPS. While oxygen supplementation caused her dyspnea to improve and oxygen saturation to rise to a safe level, she interestingly was never able to reach a saturation of 100%. However given this improvement in her dyspnea and oxygenation, as well as the resolution of all signs and manifestations of the bronchiectasis flare that she initially presented with, the patient was discharged home with oxygen. Soon after discharge, she was seen in a pulmonology clinic where she was found to be in stable condition.

**Table 1.** Patient’s orthodeoxia as established via arterial blood gas analysis.

Parameter	Supine	Seated
FiO _2_	21% (Room Air)	21% (Room Air)
PaO _2_, Arterial	65.5 mmHg	53.1 mmHg
Direct O2 Saturation	93.3%	88.6%
PaCO _2_, Arterial	45.0 mmHg	43.1 mmHg
A-a gradient	28.0 mmHg	42.8 mmgHg

FiO _2_– Fraction of inspired oxygen, PaO _2_– Partial pressure arterial oxygen, PaCO _2_– Partial pressure arterial carbon dioxide, A-a gradient – Alveolar-arterial gradient.

## Discussion

Our patient possessed all three of the cardinal findings of hepatopulmonary syndrome: chronic liver disease, hypoxemia, and evidence of pulmonary microvascular abnormalities. Her symptom of platypnea (shortness of breath worsened by going from a supine to seated position) and finding of orthodeoxia on arterial blood gas analysis also clearly pointed to HPS. But while most cirrhotic patients with hepatopulonary syndrome have only mild disease and its severity is often proportional to that of their cirrhosis, she presented with severe disease despite seemingly having compensated cirrhosis ^3^. Moreover, it is very unusual for HPS to be the first symptomatically manifesting sequela of cirrhosis as it was in this patient; there are few other examples of this happening in the literature ^4^.

As it is classically described, our patient's hypoxemia likely resulted from intrapulmonary vascular abnormalities causing ventilation-perfusion mismatch (primarily) and direct arteriovenous communications (to a lesser degree), which yielded her dyspnea ^5, 6^. More specifically this shunting caused her to have platypnea, which is the symptomatic manifestation of orthodeoxia, and has been shown to be very closely tied with HPS. In one prospective study comparing cirrhotics with HPS vs. those without the complication, platypnea was endorsed by 65.5% of the HPS group vs 6.2% of the non-HPS cirrhotic group ^7^.

Given that they are also manifestations of cirrhosis-related vascular malformations, spider angiomas are also commonly seen in HPS as they were with our patient ^8^. The intrapulmonary anomalies can be reliably detected via saline-enhanced transthoracic echocardiography, as well as with more advanced confirmatory tests such as technitium-99m macroaggregated albumin (MAA) nuclear scanning or pulmonary angiography ^3, 5, 9^. Given the clear presence of lately-transmitted bubbles seen on her transthoracic echocardiogram though, these more advanced and expensive tests were not pursued in our patient's case.

Once the diagnosis is made with the aforementioned triad, disease severity is assessed via PaO _2_. Patients with mild disease have a PaO _2_ ≥ 80mmHg, those with moderate have PaO _2_ ≥ 60 < 80 mmHg, severe have PaO _2_ ≥ 50 < 60 mmHg, and those with very severe disease have a PaO _2_ of < 50 mmHg ^10^. Our patient was found to have severe disease based upon her PaO _2_, although much of her arterial hypoxemia was likely related to her other pulmonary diseases. The pathogenesis of HPS is thought to involve increased serum levels of nitric oxide (although correlations with elevated carbon monoxide and tumor necrosis factor α have also been seen) resulting from cirrhosis, which is postulated to cause pulmonary vascular dilatation, and to a lesser degree arteriovenous malformations (AVMs) ^5, 11^. Autopsy studies have shown that the number of dilated precapillary and capillary vessels in the lungs far outnumbers the number of pulmonary AVMs in these patients, but the end result of each is the same: passage of mixed-venous blood into the pulmonary veins, resulting in V/Q mismatch and hypoxemia ^5^. Where those vessels dilated as a result of HPS are concerned, the hypoxemia is a result of diffusion-limited gas exchange. Cirrhosis itself (independent of HPS) is associated with impaired autoregulation of pulmonary vascular tone in a heterogenous distribution throughout the lung, which in HPS results in orthodeoxia as alveoli in dependent areas see alterations in ventilation for which blood flow cannot be adequately accommodated for with respect to gravitational changes when patient position is adjusted ^5, 6, 12^.

The only definitive treatment for HPS is liver transplantation ^13^. While those with this condition are more prone to post-operative complications than other patients post-liver transplant, ultimately those who survive have resolution of the hypoxemia caused by their pre-transplant HPS ^14^. Given her age, comorbidities, and otherwise well-compensated liver disease, liver transplant was not considered in our patient.

In addition to HPS, there are other similar clinical entities which are worth discussing as part of the differential diagnosis for patients who present like ours. Similar to HPS, portopulmonary hypertension (PPH) is also a sequela of cirrhosis which is characterized by pulmonary hypertension (as defined by elevated mean arterial pressure of >25mmHg at rest, and pulmonary vascular resistance greater than 240 dynes/sec/cm ^-5^) in the setting of portal hypertension and/or cirrhosis with other causes of PH having been excluded ^10^. Patients with PPH typically present with clinical findings consistent with other causes of pulmonary hypertension, namely external dyspnea, fatigue, chest pain, and/or syncope, with progression to *cor pulmonale* in severe cases ^15^. PPH was excluded in our patient by the fact that her transthoracic echocardiogram showed no evidence of pulmonary hypertension.

Hereditary hemorrhagic telangiectasia (HHT) is a genetic (autosomal dominant) disease in which arteriovenous malformations (AVMs) and mucocutaneous telangiectasis form throughout the body ^16^. The predominant symptom with which people present is paroxysmal epistaxis related to AVMs in the nasal mucosa, although 15–35% of patients with HHT have pulmonary AVMs which cause dyspnea in a manner analogous to those with HPS ^17^.

While it was tempting at first to presume that our patient's untreated MAI (and/or her other lung diseases, see Table 2 below) could be causing her symptoms, the clinical presentations which result from this infection are not typically mimickers of HPS. Those with symptomatic pulmonary MAI infection typically present in one of two ways, depending on whether they have underlying lung disease or not. Those with underlying lung disease (most commonly COPD or bronchiectasis) typically have a tuberculosis-like (albeit milder) presentation: chronic cough, weakness, malaise, weight loss, dyspnea, and upper-lobe predominant infiltrates and/or cavitation on chest imaging ^18^. Patients with underlying bronchiectasis will usually have their MAI infection develop in bronchiectatic areas, not necessarily in the upper lobes ^19^. Those without underlying lung disease tend to present with months of productive cough, without the other “tuberculosis-like” constitutional symptoms ^20^. A subset of these patients classically present with “Lady Windermere syndrome:” lingular/right-middle lobe infiltrates in elderly women without predisposing lung disease who suppress their cough ^21^. Regardless of which way a patient presents, diagnosis is made via the combination of radiographic findings indicative of pulmonary disease along with either MAI-positive sputum or an MAI-positive bronchial wash in a patient with respiratory symptoms, according to the Infectious Disease Society of America and the American Thoracic Society ^22^.

**Table 2.** Comparing notable clinical and diagnostic characteristics of her different lung disorders.

Parameter	COPD	Bronchiectasis	MAI	HPS	PPH	HHT
Productive Cough	Yes	Yes	Yes	No	No	No
Shortness of Breath with Exertion	Yes	Yes	Yes	Yes	Yes	Sometimes (if pulmonary involvement)
Shortness of Breath at Rest	Yes	Yes	Yes	Yes	Yes	Sometimes (if pulmonary involvement)
Platypnea/Orthodeoxia	No	Sometimes (if basilar predominant)	Sometimes (if basilar predominant)	Yes	No	Sometimes (if pulmonary involvement)
Pulmonary Hypertension	Sometimes (WHO Group 3)	Sometimes (WHO Group 3)	Sometimes (WHO Group 3)	No	Yes	Sometimes (Usually WHO Group 2)

COPD- Chronic obstructive pulmonary disease, MAI - mycobacterium avium-intracellulare infection, HPS – Hepatopulmonary syndrome, PPH – Portopulmonary hypertension, HHT - Hereditary hemorrhagic telangiectasia, WHO - World Health Organization.

The patient’s lack of basilar-predominant nodularities on CT scan made MAI the unlikely cause of her platypnea and/or orthodeoxia. As Table 2 also illustrates though, many of her signs and symptoms could be ascribed to her COPD and/or bronchiectasis. Obstructive airway diseases such as these are a known cause of ventilation-perfusion defects similar to those seen in HPS, as the distribution of alveolar ventilation is increased whereas that of pulmonary blood flow is not, with this effect positively correlated with disease severity but significantly hastened during acute exacerbations ^23^. However neither of these conditions are known to cause orthodeoxia, and her COPD was stable (FEV ^1^ was 0.61 liters on recent spirometry) at the time of her hospitalization. In the end, the clinical and diagnostic characteristics of HPS are so specific as a result of its entirely unique gas-exchange pattern that it can be reliably diagnosed even in the setting of other diseases that cause arterial hypoxemia, as it was in our patient ^24^.

Hepatopulmonary syndrome belongs on the differential diagnosis for dyspnea in any patient with chronic liver disease, even those like our patient without any previous decompensations of cirrhosis. While it can present in a way which is symptomatically similar to that of other chronic lung diseases, it can be definitively diagnosed via a relatively simple work-up which should be performed in any dyspneic patient with evidence of liver disease. While the only definitive cure is liver transplant, it can be conservatively managed via oxygen therapy when transplant is contraindicated, as it was in our patient's interesting case.

## Consent

Written informed consent for publication of their clinical details and/or clinical images was obtained from the patient.

## Data availability

All data underlying the results are available as part of the article and no additional source data are required.
