# Supplementary material for: Case Report: Hepatopulmonary syndrome as the first clinical manifestation of cirrhosis in a patient with underlying chronic lung disease
Source: F1000Res. 2019 May 16;7:1175. Originally published 2018 Aug 2. [Version 2] doi: 10.12688/f1000research.15434.2 (PMC6635985; doi:10.12688/f1000research.15434.2)
Supplement: Peer review history

# Peer review: Reviewer response for version 2

**DOI:** 10.5256/f1000research.21081.r50999

**Published:** 2019-07-15

**Authors:** Elias Kouroumalis

**Affiliations:** 1 Department of Gastroenterology, Medical School, University of Crete, Heraklion, Greece

This is a case report of a complicated situation. An old lady with many chronic pulmonary problems was presented with what was considered at first as an ordinary lung infection complicating her COPD and/or bronciectasis. Despite antibiotic treatment her dyspnea was not improved despite improvement of inflammatory indices. This led to an additional work up, revealing the existence of an unsuspected cirrhosis with HPS. 

 The presentation is informative, well written, the diagnosis of HPS is fairly well documented and the discussion is very informative on the differential diagnosis of HPS. My reservation is that the authors classify their patient as having severe HPS based on PaO2 measurements. Although they accept that the other confounding comorbidities may influence the results, the subsequent progress of the patient seem to condradict their conclusion. Severe HPS is not expected to lead to an uneventful course after treatment of the other comorbidities. I feel that this should be commented in the discussion.

I confirm that I have read this submission and believe that I have an appropriate level of expertise to confirm that it is of an acceptable scientific standard.

# Peer review: Reviewer response for version 2

**DOI:** 10.5256/f1000research.21081.r48759

**Published:** 2019-07-10

**Authors:** Mohan Rudrappa

**Affiliations:** 1 Department of Pulmonary and Critical Care Medicine, Mercy Hospital, Joplin, MO, USA

Well written article.

 The case presentation is good with attention to the relevant symptoms.

 The management strategies are also excellent. 

 The discussion part is well written and the authors have considered all the potential causes and have systematically and reasonably rule out other causes.

 It would be ideal if the authors upload the video of the bubble study and specify after how many cycles they saw the shunt just to rule out intracardiac shunt. Any intracardiac shunt can produce the triad of hypoxia, orthodeoxia and platypnea

I confirm that I have read this submission and believe that I have an appropriate level of expertise to confirm that it is of an acceptable scientific standard.

# Peer review: Reviewer response for version 1

**DOI:** 10.5256/f1000research.16819.r37169

**Published:** 2018-08-29

**Authors:** Robert Rodríguez-Roisin

**Affiliations:** 1 Pneumology Service, Thorax Institute, Hospital Clinic-The August Pi i Sunyer Biomedical Research Institute (IDIBAPS)-Networked Centre for Biomedical Research on Respiratory Diseases (CIBERES) (Hospital Clínic-IDIBAPS-CIBERES), University of Barcelona, Barcelona, Spain

This is another case report of an interesting and complex HPS in the context of several comorbidities, such as COPD and bronchiectasis, let alone the coexistence of MAI infection, that each, all in combination or even one more than another entity, can cause arterial hypoxaemia, along with an incomplete differential diagnosis approach. All in all both the discussion and presentation are weak and modest which detract from the potential clinical interest of the case report. Behind this negative short review lie three major caveats.

 First, the lack of a better knowledge on gas exchange abnormalities in HPS is overwhelming despite that some of the quoted references (i.e., ref 7) masterly describe the pathophysiology of gas exchange alterations in HPS. Yet, the Authors miss a succinct good description. One of the reasons of this is that the Author is confounded by the use of the broad term of ‘shunting’ to encompass the three well established intrapulmonary determinants of arterial hypoxaemia in HPS (ventilation-perfusion imbalance, increased intrapulmonary shunt, and diffusion limitation to O2 transfer) quite thoroughly reported in a recent publication of the Amer Physiol Soc (Rodriguez-Roisin R, et al. ^1^). May I therefore suggest to the Author to take a look at this publication and make the appropriate changes in your paper. HPS is not a problem of ‘intrapulmonary shunting’, as it is repeatedly quoted, but a predominant ventilation-perfusion mismatching along with other additional altered pulmonary and non-pulmonary determinants of gas exchange (see also, Rodriguez-Roisin ^2^).

 Second, the Author does not afford the lung function testing of this lady, including the most likely smoking habits of his old patient which are completely ignored. As a result, we are in the middle of nowhere to grasp the comprehensive nature of her gas exchange abnormalities. Are these due to her severe chronic pulmonary problems (COPD with bronchiectasis), to HPS as the author is inclined to, without providing any rationale, or to a combination of both pulmonary and liver disorders (most likely). The lack of a proper differential diagnosis is another of the main gaps. I, for me, suggest to also read a very interesting paper focusing on cardiopulmonary comorbidities and HPS (Martinez GP, et al. ^3^) that may help the Author to improve his differential diagnosis. Moreover, the Author has completely neglected the relevant role of the alveolar-arterial oxygen partial pressure difference. With due respect, I suggest you read again ref 7, now more critically and more in detail!

 Third, I have to admit that although it is most likely that the arterial hypoxaemia of this old lady is due to a combination of lung and hepatic problems, there is a sentence in the Case Report that worries me. It says: ‘Soon after discharge, she was seen in a pulmonology clinic where treatment for MAI was commenced. When last seen a month afterward, the patient reported that she was tolerating her treatments well and felt improved since her hospital discharge’. This sentence makes me suspicious and questions me if the hypoxaemia is due to HPS for there are no pharmacological options for HPS so far.

I confirm that I have read this submission and believe that I have an appropriate level of expertise to state that I do not consider it to be of an acceptable scientific standard, for reasons outlined above.

## References

[1] : Hepatopulmonary Disorders: Gas Exchange and Vascular Manifestations in Chronic Liver Disease. *Compr Physiol*.2018;8(2) : 10.1002/cphy.c170020 711-729 10.1002/cphy.c170020 29687908

[2] : Nonpulmonary influences on gas exchange. *Compr Physiol*.2014;4(4) : 10.1002/cphy.c100001 1455-94 10.1002/cphy.c100001 25428851

[3] : Hepatopulmonary syndrome associated with cardiorespiratory disease. *J Hepatol*.1999;30(5) :882-9 1036581610.1016/s0168-8278(99)80143-3
